# Scaling up health policies and services in low- and middle-income settings

**DOI:** 10.1186/1472-6963-10-S1-I1

**Published:** 2010-07-02

**Authors:** Kara Hanson, Susan Cleary, Helen Schneider, Sripen Tantivess, Lucy Gilson

**Affiliations:** 1 Department of Global Health and Development, London School of Hygiene and Tropical Medicine, UK; 2 Health Economics Unit, University of Cape Town, South Africa; 3 Centre for Infectious Diseases Epidemiology and Research, University of Cape Town, South Africa; 4 Health Intervention and Technology Assessment Program, Ministry of Public Health, Thailand; 5 School of Public Health and Family Medicine, University of Cape Town, South Africa

## 

“Scaling up” effective health services is high on the policy agendas of many countries and international agencies. The current concern has been driven by growing recognition both of the challenges of achieving the health-related Millennium Development Goals (MDGs) in many countries, and of the need to ensure that the increased resources for health channelled through disease-specific health initiatives are able generate health gain at scale.  Effective and cost-effective interventions exist to address many of the major causes of disease burden in the developing world, but coverage of many of these services remains low. There is a substantial gap between what could be achieved and what is actually being achieved in terms of health improvement in low- and middle-income countries.  

The term “scaling up” is widely used as shorthand to describe the objective or process of expanding service delivery. As this broad definition suggests, the term scaling up has been used in a variety of ways and contexts. For example, it can refer to the *outcome*, in terms of increased coverage, or the *inputs* required, whether financial, human or capital resources. Similarly, scaling up can also refer to a *policy*, *strategy* or the *process* of expansion [1]. The object of scaling up can be particular health interventions (e.g. attended delivery, insecticide treated mosquito nets, or integrated management of childhood illness), or health systems interventions such as health financing mechanisms (community-based health insurance), incentive mechanisms (e.g. pay-for-performance or contracting) or approaches to service delivery (e.g. training shopkeepers to supply antimalarial drugs). 

A recent review article [1] and a set of accompanying commentaries [2-4] documented the origins and use of the term scaling up in international health, and identified a number of critical themes or issues in scaling up health policies and interventions: 

- the cost of scaling up and resources required to expand service delivery , including the trade-offs that may arise between equity and efficiency

- the constraints to scaling up that operate at different levels, from the household and community level through to the service delivery, strategic and national policy, and cross-sectoral levels

- the potential synergies and deleterious health system effects of global health initiatives

- the opportunities afforded by novel approaches to service delivery, including making use of private sector delivery channels.

However, despite increased conceptual clarity in this area, there remains relatively little empirical evidence on these themes.  In June 2009 the Consortium for Research on Equitable Health Systems (CREHS – http://www.crehs.lshtm.ac.uk), a research programme funded by the UK Department for International Development which brings together 8 health policy and systems research groups from 7 countries, hosted a writing workshop on the theme of Scaling Up Health Policies and Interventions. Workshop participants, drawn from CREHS members and collaborators, presented and discussed research they had been conducting across a variety of programmatic areas linked to this theme. The eight papers in this supplement represent a selection of the papers presented at this workshop. Together, they contribute a rich set of new evidence about the barriers to scaling up, the opportunities for overcoming these through changes in financing arrangements and service delivery innovations, and the critical importance of the processes of managing change in order to realise the promise of scaled up programmes and interventions. 

The papers in this collection address three of the priority health areas identified in the MDGs: antiretroviral therapy for the treatment of HIV/AIDS, reproductive health services (cervical cancer screening), and malaria treatment. In addition, the first and last papers have a generic, rather than disease-specific focus, one examining issues of attracting and retaining health workers in rural areas of Kenya, and the other proposing an approach to evaluating the impact of novel service delivery strategies. 

Building on shared programmatic concerns, four other important themes emerge from the papers: 

###  (1) Costs and resource needs

There is a substantial literature estimating the cost of scaling up individual interventions or packages of interventions, most recently represented in the report of the Task Force on Innovative International Financing for Health Systems [5]. Costing ARV scale-up is substantially more complicated than other programmes because once an individual begins ART, they must keep taking the medicines for the rest of their life. The lifetime costs of ART must therefore be modelled based on assumptions about not just the cost of first line therapy and the number of new infections, but also the effects of ART on longevity, the likelihood of needing to switch to second line drugs, and the other health care costs that are incurred such as management of opportunistic infections.  The paper by Leisegang and colleagues [6] adds to the literature on modelling the costs of scaling up ART by analysing the variables that are associated with different health outcomes and health care costs, using data from a large cohort of individuals receiving ART in South Africa.

Few costing studies make any serious attempt to address the question of how these costs are to be financed, or the appropriate contributions of domestic vs. external financing. Cleary and McIntyre [7] link their estimate of the cost of scaling up ART in South Africa to proposals to reform the South African health financing system to develop a universal coverage scheme based on a combination of contributory mandatory insurance and increased tax funding. A key insight from their paper is the relationship between ART access and the broader policy context, noting that only by addressing the fundamental inequities in the health financing system will it be possible to achieve equitable, affordable and sustainable ART access.

The shortage of human resources in many low-income settings, particularly in rural areas, is a major constraint to expanding service coverage. Mullei and colleagues [8] examined the perceptions and attitudes of Kenyan nursing school graduates towards working in rural areas in order to identify policies that might encourage health workers to locate to these underserved areas. They found that nursing students hold a variety of positive and negative attitudes towards working in rural areas. As well as investments in higher salaries and improved infrastructure, respondents identified changes in organizational arrangements, including the ability to choose their location, opportunities for more rapid career advancement, and improvements in contractual conditions, as policies that could contribute to a more equitable distribution of health workers.

### (2) Managing policy processes

Another scaling up challenge is the need for the related policy development and implementation processes to be managed strategically to support service expansion. Three papers in this supplement address strategic management. Yothasamut et al. [9] consider both how screening for cervical cancer was adopted, over the human papilloma virus vaccine, as the preferred cervical cancer prevention strategy in Thailand and how the imperative of a political opportunity generated both opportunities and challenges for its scaling up. Abuya et al. [10] and Schneider et al. [11] focused, respectively, on training programmes to improve malaria treatment provided by shopkeepers in Kenya and on ART roll-out in South Africa, both carefully examining implementation processes. Yothasamut et al. reflect a wider body of evidence in showing how the agenda setting phase of policy development is often influenced by contestation between national and international policy actors supporting different policy options, evidence around those options and the timing of these debates in relation to a national political window of opportunity [12]. That opportunity, a government-wide programme of action, spurred on implementation but also precluded the planning and preparation needed to support achievement of scaling up goals. Abuya et al., meanwhile highlight the local level action and learning needed to support effective scaling up of an innovative public health intervention, and the need for deliberate but flexible management strategies that respond to both unexpected events and the more predictable tensions among implementing actors. Finally, Schneider et al. illustrate the importance of sub-national political and managerial support and leadership for scale up; the need to balance standardisation with programme flexibility; and the significance of “clinical” partnerships and monitoring and evaluation systems.

### (3) Private sector

Growing recognition of the role played by the private sector in malaria treatment has led to interest in how to improve the quality of treatment in this sector.  Two papers in this supplement address the use of the private sector to deliver malaria medicines. Abuya et al. describe three different projects which aimed to improve the quality of malaria treatment in the informal sector through shopkeeper training. Because they involve multiple partners (private sector, local government or NGO implementers, and technical teams which often come from outside), these approaches raise particular issues of strategic management. Cohen et al. [13] examine a small scale programme pilot-testing an innovative mechanism for subsidising artemisinin-based combination therapy (ACTs) delivered through private drug shops in Tanzania. The paper focuses on how distance mediates the effect of price subsidies on both the decision by shops to stock these new drugs and on sales volumes. The paper demonstrates both the potential for the private sector to increase coverage of effective malaria treatment, and the need to be ever-vigilant that such approaches address the needs of the poor. The results of this project have fed directly into the decision to pilot this approach at a national scale in 8 countries through the Affordable Medicines Facility – malaria (AMFm), managed by the Global Fund. 

### (4) Methods for studying scaling up

Webster et al. [14] review the literature evaluating service delivery systems for malaria related interventions, including insecticide-treated nets and intermittent preventive treatment in pregnancy. They reflect on the complexity of these systems and indicate that evaluation needs to look comprehensively at the multiple steps in the delivery process and at the multiple factors influencing it. Their proposed method for evaluating service delivery arrangements could be achieved through the insertion of questions in routine household surveys about the location and mechanism of service provision to aid in attribution of coverage outcomes to specific delivery systems.

The collection as a whole also points to the need to develop and refine the methodology of health policy and systems research for addressing scaling up. The papers adopt a range of study designs and analytical approaches – cross-sectional surveys, Markov and financial modelling techniques, case studies using a combination of document review and qualitative data framed through theory, and impact evaluation using a quasi experimental design. The main conclusion from these studies is that scaling up processes are complex. Evaluations should therefore not be limited to programme impact, but must include a way of understanding and interpreting the processes through which new activities are implemented within the health system [10,11], and the processes through which new ideas become policies [9]. More broadly, the studies support calls for new evaluation approaches that take account of the complexity of health interventions. De Savigny and Adam [6,15] for example, have called for the application of “systems thinking” approaches both in designing interventions and evaluating them. They propose that evaluation should include process evaluation (for adequacy), context evaluation (for transferability), effects evaluation (looking at both intended impacts and unintended consequences), and economic evaluation (to address cost-effectiveness considerations).  Drawing on wider thinking and experience around evaluating complex social programmes, these components also recognise the importance both of drawing multiple perspectives into the development and evaluation of large scale interventions, and of evaluation study designs that recognise multiple pathways to outcomes and the likelihood of unexpected consequences.

These papers are published at a critical time for health systems: while some countries are on track to achieve the 2015 MDG targets, others are faltering and action is needed now to accelerate progress. Important gaps in knowledge remain. In particular, our understanding of both policy development and service delivery strategies in fragile states is limited, and there is scope for research on how to most effectively scale up services in these environments. Perhaps most critical is the recognition that any scaling up process must include both opportunities to learn through action and a way to feed the lessons of experience into strategies to strengthen implementation. 

## Competing interests

The authors declare that they have no competing interests.
